# Highly Aggressive CD4-Positive Mast Cell Leukaemia (Leukaemic Variant) Associated with Isolated Trisomy 19 and Hemophagocytosis by Neoplastic Mast Cells: A Case Report with Challenging Experience and Review

**DOI:** 10.1155/2019/1805270

**Published:** 2019-10-27

**Authors:** Dina Sameh Soliman, Ahmad Al-Sabbagh, Feryal Ibrahim, Amna Gameil, Mohamed Yassin, Halima El-Omri

**Affiliations:** ^1^Department of Laboratory Medicine and Pathology, National Center for Cancer Care and Research, Hamad Medical Corporation, Doha, Qatar; ^2^Clinical Pathology Department, National Cancer Institute, Cairo University, Cairo, Egypt; ^3^Clinical Pathology and Laboratory Medicine, Weill-Cornell Medicine, Doha, Qatar; ^4^Department of Hematology and Medical Oncology, National Center for Cancer Care and Research, Hamad Medical Corporation, Doha, Qatar

## Abstract

**Background:**

Mast cell leukaemia is a unique disease among hematopoietic neoplasms, being one of the rarest leukaemia subtypes. In addition, its prompt diagnosis is usually challenging. This is due to its heterogeneity in clinical presentations and cytomorphological and immunophenotypical features together with potential associations with other hematologic neoplasms which can complicate the condition and delay accurate diagnosis. To the best of our knowledge, this is the first case report of CD4-positive mast cell leukaemia.

**Case Presentation:**

A 39-year-old male presented with acute onset of fever, abdominal pain, and generalized body aches of two-week duration. Peripheral blood smear showed circulating blasts (13%) with coarsely basophilic granulation. Bone marrow (BM) aspirate showed extensive infiltration with immature mast cells of blast-like morphology with trilineage dysplasia and evident hemophagocytic activity exhibited by histiocytes and neoplastic mast cells. BM biopsy was diffusely infiltrated with many atypical mast cells positive for CD45, CD117, mast cell tryptase, CD25, and CD4 with partial positivity for CD7 and CD30. Cytogenetics showed an abnormal karyotype: 47, XY, +1947, XY, +19[13]/46, XY[9]. Molecular analysis revealed a *KIT* D816V mutation consistent with a diagnosis of systemic mastocytosis, mast cell leukaemia.

**Conclusion:**

The expression of T-cell associated markers by abnormal mast cells is well documented; however, CD4 and CD7 expression have not previously been described in association with mast cell leukaemia. Coexpression of CD2, CD4, CD7, and CD30 by the mast cells particularly in skin lesions may provoke misinterpretation as a cutaneous T-cell neoplasm. To the best of our knowledge, this is the first report of CD4-positive mast cell leukaemia. Moreover, hemophagocytic mast cell leukaemia is a very rare morphologic variant, and possible correlation between this finding and expression of CD4 by neoplastic mast cells is a topic for further investigation.

## 1. Background

Mast cell leukaemia (MCL) is a very rare form of aggressive systemic mastocytosis (SM) accounting for <0.5% of all mastocytosis patients in the French Reference Center for Mastocytosis (CEREMAST), representing one of the rarest leukaemia subtypes. It is characterized by the leukaemic spread of mast cells (MCs), with frequent multiorgan involvement. The prognosis is exceedingly poor, and therapy usually fails with a median survival of less than 6 months.

According to the 2008 WHO and the most recently revised WHO classification (2016), MCL diagnosis is based on the presence of >20% atypical/immature MCs in the BM smears and/or >10% in the blood in addition to fulfilment of the WHO diagnostic criteria of SM [1].

The case described here showed a unique pathologic feature with exceptional immunophenotypic profile being a de novo leukaemic variant with atypical expression of CD4, CD7, and mast cells exhibiting erythrophagocytosis.

Although high suspicion index based on morphologic characteristics was the clue to the diagnosis, the main diagnostic challenges were the nonspecific clinical and pathological features, absence of symptoms of mast cell activation, scarcity of circulating neoplastic cells at initial presentation, and expression of T-cell-associated markers on neoplastic MCs (CD25, CD4, CD7, and CD30).

## 2. Case Presentation

A 39-year-old male, with a past medical history of treated tuberculosis, presented to the emergency department complaining of fever, generalized weakness, vague abdominal pain, vomiting, headache, and generalized body aches of two-week duration with a history of nasal bleeding of moderate severity. Upon examination, there was submandibular lymphadenopathy (2 × 1 cm) and palpable liver (7 cm below the costal margin) and no gastrointestinal manifestations and skin lesions.

Laboratory workup revealed high urea level at 18.84 mmol/L (3.2–7.4), serum creatinine at 141 *μ*mol/l (64–110), lipase at 182 U/L (8–78), myoglobin at 108 ng/ml (1–78), and normal liver function tests.

Further investigations revealed very high ferritin level >40000 mcg/L (24–336), low serum B12 level (<111 pmol/L), normal fibrinogen level at 4.77 gm/L (1.8–3.5), and normal triglyceride level at 0.6 mmol/L (normal: <1.7 mmol/L). Viral serology for hepatitis B and C and HIV was negative.

His complete blood count showed pancytopenia, with hemoglobin of 6.4 gm/dL (13.0–17.0), total white blood count (WBC) of 1.7 × 10^3^/*μ*l (4.0–10.0), absolute neutrophil count (ANC) 0.9 × 10^3^/*μ*l, and platelet count of 2 × 10^3^/*μ*l (150–400). BM failure was suspected, and the patient was admitted for further workup.

Sepsis workup was requested, and the patient was started on antimicrobials and vitamin B12 replacement.

Computerized tomography (CT) scan of the head showed bilateral subdural haemorrhage with no midline shift, and the patient was managed conservatively with transfusion support. Whole body CT scan showed bilateral pleural effusion, extensive pulmonary consolidation, thrombosis of the celiac artery and its branches with splenic infarction, mild hepatomegaly, and massive splenomegaly. The spleen showed poor heterogeneous enhancement suggestive of infarction.

**Figure 1:**
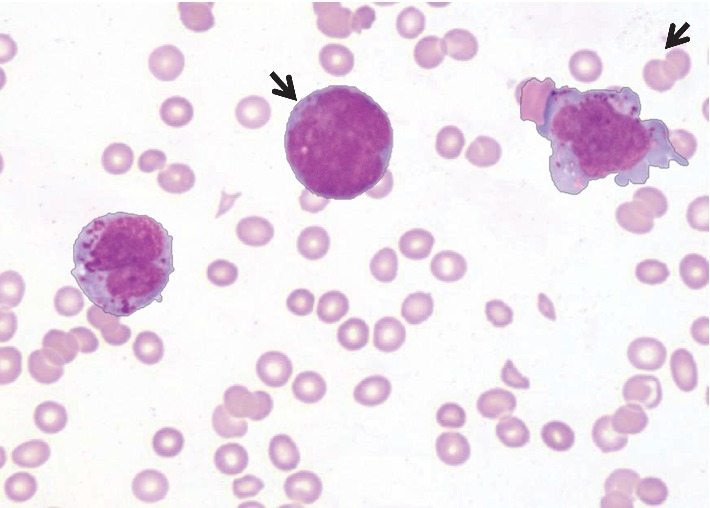
Peripheral smear showing immature cells with blast morphology (arrows) and atypical mast cells.

First, peripheral blood smear (PS) was inconclusive as it showed very few circulating atypical cells. Subsequent PS (Figure 1) showed marked leukopenia with some circulating abnormal blastoid cells (13%) of medium size with fine nuclear chromatin and unevenly distributed deeply basophilic/metachromatic coarse granules. Few blasts showed nuclear bilobation.

**Figure 2:**
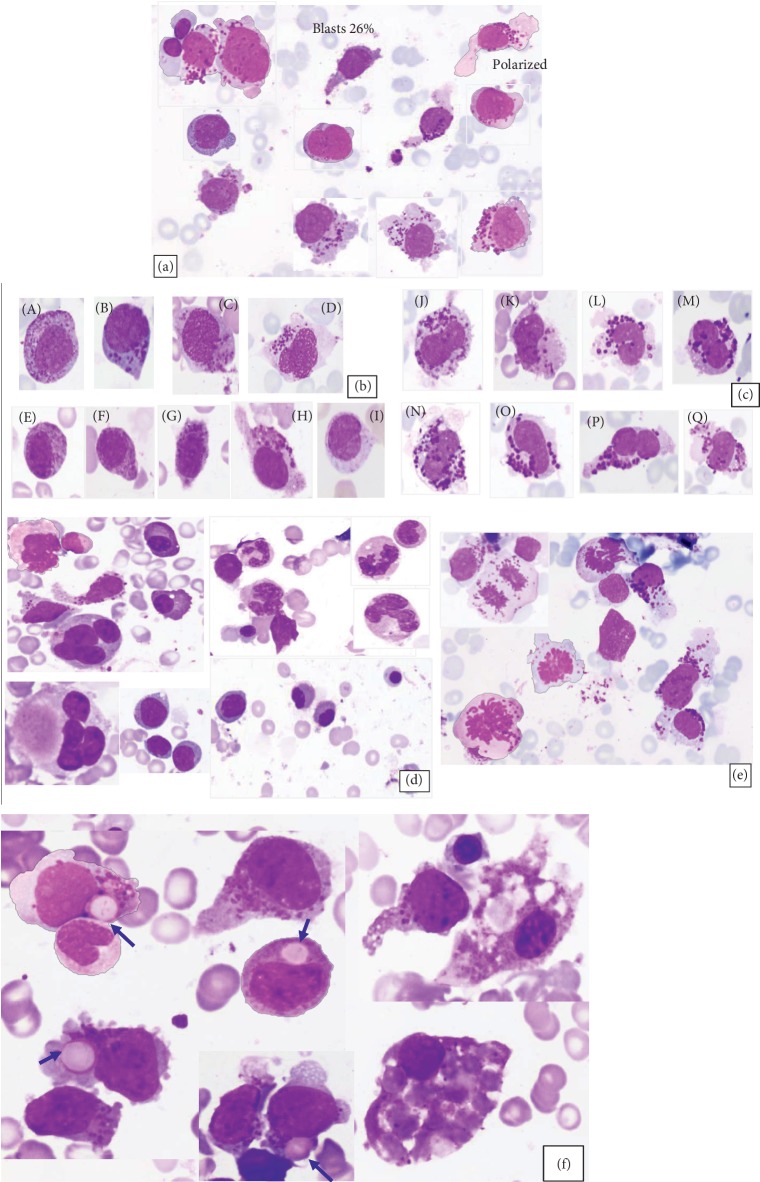
BM aspirate smear (100x) showing a variety of neoplastic MCs. The majority of blasts show abundant cytoplasm and coarse giant metachromatic blasts (a). Neoplastic MCs (Type I) (A–I) with eccentric round/oval nuclei, uneven granule distribution/hypogranulation, and spindle-shaped MCs with elongated cytoplasmic projections (b). Neoplastic MCs Type II (promastocytes) (A–H) with indented, bilobed nuclei, abundant hypogranulated cytoplasm, or with coalescent granules with uneven distribution (c). There is trilineage dysplasia with macronormoblasts, giant neutrophils, and dysplastic nuclear lobulation in megakaryocytes (arrows) (d). Increased mitotic figures (e). Prominent erythrophagocytosis exhibited by neoplastic mast cells (blue arrow) and histiocytes (f).

Bone marrow (BM) aspirate was infiltrated with numerous atypical and immature MCs of blast-like morphology (26%) showing fine nuclear chromatin and few prominent nucleoli. The majority of blasts showed eccentric nuclei with abundant cytoplasm and coarse giant metachromatic/basophilic granules (metachromatic blasts) (Figure 2(a)). Few blasts were agranular with high nucleocytoplasmic ratio and few were spindle-shaped. The majority of neoplastic MCs exhibited marked atypia and pronounced pleomorphism (Figures 2(b) and 2(c)) of (Type I) (Figure 2(b), A–I) with eccentric round/oval nuclei, uneven granules distribution/hypogranulation, and spindle-shaped mast cells with elongated cytoplasmic projections. Neoplastic MCs Type II (promastocytes) (Figure 2(c), A–H), with indented, bilobed nuclei and abundant degranulated/hypogranulated cytoplasm or with coalescent granules and uneven distribution were frequent.

There was trilineage dysplasia with prominent megaloblastic changes mostly in the form of giant granulocytes, megakaryocytes with multilobulation, and some megaloblastic erythroid precursors (composite, Figure 2(d)) with some mitotic figures noted (Figure 2(e)). There was an evident hemophagocytic activity (predominantly erythrophagocytosis) exhibited by histiocytes and neoplastic mast cells (Figure 2(f)).

**Figure 3:**
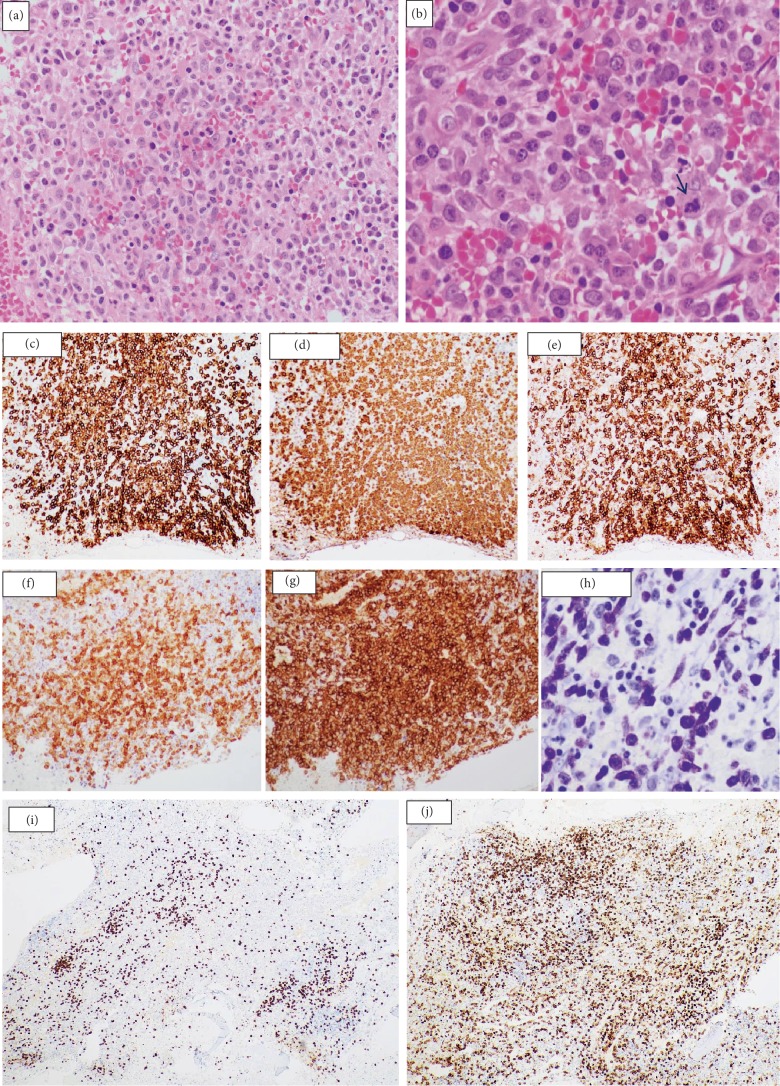
BM biopsy (H&E) showing diffuse infiltration by atypical mast cells 20x (a). 50x magnification of BM biopsy showing increased mitotic figures (arrow) and evident erythrophagocytosis (b). Immunohistochemistry on BM biopsy: the neoplastic mast cells are positive for (c) CD117, (d) tryptase, (e) CD25, and (g) CD4 and show partial positivity for (j) CD7 and (f) CD30. (i) CD3 immunostain was included to show the normal CD3-positive T cells. (h) Toluidine blue highlighted the more differentiated MCs.

On H&E, BM biopsy was markedly hypercellular, largely replaced by sheets of neoplastic mast cells showing abundant clear cytoplasm with round/indented nuclei (including many forms with immature nuclear chromatin), and admixed with a reactive background composed of fibroblasts, eosinophils, small lymphocytes, and polyclonal plasma cells (Figure 3(a) and 3(b)). There was a mild increase in reticulin fibres (MF 0-1). By immunohistochemistry (IHC)(Figure 3), the neoplastic MCs were positive for CD45, CD117, mast cell tryptase, CD25, CD43, CD4, and partially positive for CD30 and CD7 while negative for CD2. Histiocytes/macrophages were prominent with evident hemophagocytic activity. Toluidine blue and Giemsa stain highlighted the metachromatic granules of the more differentiated MCs while the majority was negative. Megakaryocytes appeared focally increased with some small hypolobated forms and some degree of morphologic atypia. Serum tryptase level revealed very a high level at 434.00 mcg/L (negative < 11 mcg/L).

**Figure 4:**
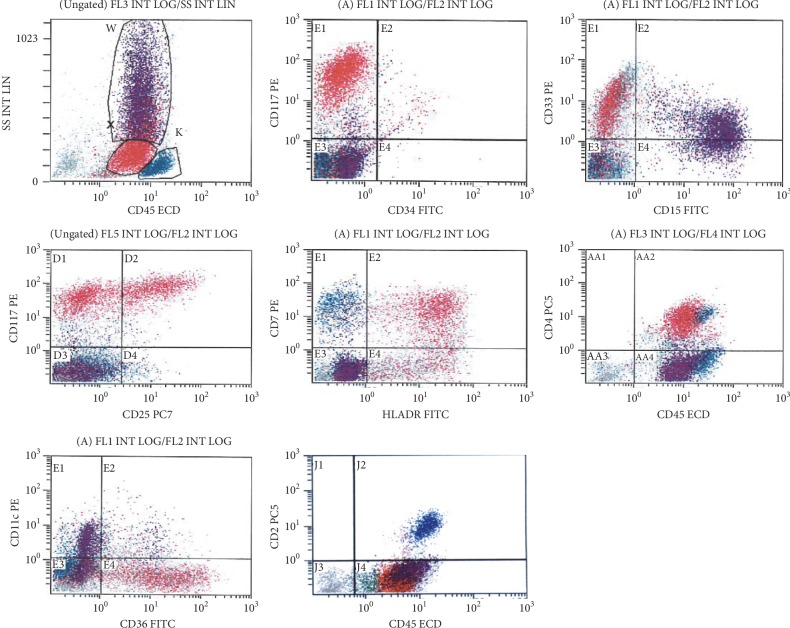
FCM shows an abnormal population of mast cells (red) in the blast gate; approximately 26% (of the total cells) expressing bright CD117, and the majority is positive for CD33 and HLADR with partial aberrant CD25 on a subpopulation (∼11% of total). The cells show aberrant expression of CD7 and CD4, partial expression for CD36, and negative for CD2.

Flow cytometry on BM (Figure 4) showed an abnormal population of cells in the blast gate comprising 25%, expressing CD45 (moderate), CD117 (bright), CD33, and HLA-DR with partial aberrant CD25 expression. The neoplastic cells showed aberrant expression of CD4, aberrant partial expression of CD7, and partial expression of CD36. In addition, the analysis showed a small population of cells (∼4%) with low side and forward light scatter expressing CD33 and CD4 with a minor subpopulation expressing CD117, CD64, and CD14, possibly representing dysplastic myeloid/hypogranular mast cells. The neoplastic mast cells are negative for cMPO, CD64, CD14, CD19, CD20, cCD3, sCD3, CD5, CD2, CD11c, and CD11b.

Cytogenetic studies showed an abnormal karyotype with isolated trisomy 19 (47, XY, +19[13]).

Molecular analysis of blood confirmed a *KIT* D816V mutation with negative *BCR/ABL-1*, consistent with a diagnosis of systemic mastocytosis, mast cell leukaemia.

The patient developed acute kidney injury with progressive deterioration in renal function (serum creatinine at 245 *μ*mol/L) and disseminated intravascular coagulopathy (DIC). Echocardiography showed severe pulmonary hypertension with normal systolic left ventricular function (ejection fraction of 60%).

On the 3^rd^ day after hospitalization, he developed respiratory distress and was started on noninvasive pressure ventilation (NIPV), diuretics, and broad-spectrum antibacterial, antifungal therapy with steroid along with supportive measures after bone marrow results.

The patient developed septic shock with multiorgan failure, so he underwent hemodialysis and was started on prednisone but unfortunately developed cardiac arrest (asystole) and died two weeks after admission.

## 3. Discussion and Conclusion

Since 1950, there are fewer than 70 well-documented cases of MCL reported to date; 51 MCL cases were summarized in the largest review published in *Blood* in 2013 [2]. MCL shares more clinical and biologic features with aggressive systemic mastocytosis (ASM) than with acute myeloid leukaemia (AML) [3].

When considering a diagnosis of MCL; it is important to distinguish the following subvariants of MCL : 1, primary (de novo) MCL versus secondary MCL, the latter is preceded by another subtype of mastocytosis, commonly evolution form ASM and 2, “Pure” MCL versus MCL associated with hematological neoplasm (MCL-AHN). The majority of MCL cases are de novo (73%), i.e., not preceded by chronic mastocytosis and not associated with hematologic neoplasms. More recently, MCL has been divided into a chronic form without obvious organ damage (no C-findings present) and a more aggressive (acute) variant, termed acute MCL where organ damage (C-findings) is present [4].

No difference was found in overall survival between de novo MCL vs. secondary MCL (*P*=0.9) or MCL-AHN vs. MCL (*P*=0.2) [5].

“Aleukaemic variant” of MCL (with circulating MCs <10%) is the most frequently described form accounting up to 62% of 52 cases reported by Georgin-Lavialle et al. [2]. MCL with more than 10% circulating MCs “leukaemic variant” (as reported here) is a rare presentation, reported in 2 out of 28 cases of MCL (7%) [5].

### 3.1. Challenging Diagnosis and Differential Diagnosis

This case fulfilled the diagnostic criteria for SM with the presence of multifocal dense infiltrates of MCs (>15 MCs in aggregates) detected in BM sections (a major criterion for SM) in addition to all 4 minor criteria: (a) >25% of the MCs in the BM infiltrate has atypical morphology and >25% of all MCs in BM aspirate smears is immature or atypical, (b) detection of an activating point mutation at codon 816 of *KIT* in the blood, (c) the aberrant expression of CD25 in addition to normal MC markers, and (d) very high serum total tryptase (>20 ng/ml) in absence of an associated myeloid neoplasm. We classified the case as de novo MCL; a leukaemic variant with acute onset, as there was no previous history of mast cell disease or other hematologic neoplasms, no history of skin manifestations, and no history of long-standing cytopenias.

The major differential diagnosis included the following: acute basophilic leukaemia, tryptase-positive AML, and blastic plasmacytoid dendritic neoplasms (CD4/CD56-positive). However, the characteristic morphology, very bright CD117 and negativity for CD34, excludes AML. Immunophenotypic detection of abnormal MCs expressing very bright CD117, mast cell tryptase, and CD25 distinguish MCL from acute basophilic leukaemia and blastic plasmacytoid dendritic neoplasms.

This case did not show any of the mediator release symptoms caused by mast cell activation/degranulation that include severe hypotension, syncope, anaphylaxis, flushing, headache, purities, or gastrointestinal symptoms. This is in line with what was reported by Georgin-Lavialle et al. [2], who concluded that symptoms of MC activation are more frequent among MCL patients without *KIT* D816V mutation than those in the group with this mutation. Skin involvement that is usually seen in patients with indolent SM is less frequently detected in ASM and is rarely seen in MCL [4].

The diagnosis of MCL is usually challenging because of heterogeneous clinical and morphological features. In general terms, MCs are not readily recognized on BM sections and commonly confused with a variety of other cells that include fibroblasts, histiocytes, hairy cells, and monocytes. Besides, the lack of a specific MC marker also hampers prompt identification and characterization of MCs in BM.

In our case, the presence of circulating atypical cells with the characteristic coarse metachromatic granules was the first clue in making the diagnosis. Morphologically, the neoplastic MCs were so pleomorphic and showed a spectrum of maturation; however, with many metachromatic blasts mixed with numerous more differentiated neoplastic MCs (Type I and Type II) and few well-differentiated MCs. Mitotic figures were frequently seen reflecting a high proliferative index. Mitoses are rarely seen in mast cell neoplasms even in aggressive forms of mastocytosis, except in patients with MCL [5]. Interestingly; some MCs exhibited pronounced hemophagocytosis, a unique and extremely rare finding described in MCL in which highly atypical mast cells exhibit erythrophagocytosis. “Hemophagocytic” MCL is not, however, regarded as a distinct subtype of MCL [6, 7].

The significance of this morphology and its relation to disease aggressiveness are unknown.

### 3.2. Immunophenotype of MCs and MCL

Immunophenotypically, it is widely accepted that normal and neoplastic MCs express mast cell tryptase, CD117 (bright), CD45, CD33, CD11c, CD43, and CD68, but lack the expression of the myeloid markers (CD14, CD15, and myeloperoxidase) and B-cell-related antigens [1].

The neoplastic MCs usually show high light side scatter and almost always express tryptase and CD117 (bright). Other frequently expressed markers on abnormal MCs include CD25, CD2, and CD30. Flow cytometry is quite sensitive in detecting MCs even in a low percentage as they usually have a very bright CD117 expression. Because of the low frequency of MCs in BM samples, most data about the normal maturation patterns of MCs are based on in vitro differentiation studies [8].

Recent reports suggest that the combined use of only two markers (CD117 and CD45) would be sufficient to identify MCs appropriately in BM (CD117high/CD45low) [9].

Although expression of CD2 and/or CD25 is the most common immunophenotypic aberrancies on MCs and is acknowledged as a minor criterion for the diagnosis of mastocytosis, about one-third of MCL cases reported in the literature had a double-negative CD2/CD25 immunophenotype [2]. Normal well-differentiated MCs usually do not express CD2 or CD25 [10].

The association between *KIT* D816 mutation and dual expression of CD2/CD25 on MCs in SM is very frequent; dual coexpression of CD2 and CD25 was found in 66% of *KIT* D816-positive MCL patients compared with those who were *KIT* D816 negative (25%) [2].

CD25+/CD2− MCs seen in our case are a rare occurrence in MCL and are reported in 13% of MCL cases reviewed by Georgin-Lavialle et al.; CD25 and CD2 expressions varied; 13% were CD25+/CD2−; 54% of patients were double positive (CD25+/CD2+); 38% were CD25−/CD2− double negative; and 4% were showing CD25−/CD2+ phenotype [2].

Compared to patients with indolent SM, the number of “CD2-positive” MCL patients is low, and if detected, the levels are usually lower than those found on MCs in indolent SM [11].

It has been recently proposed that aggressive forms of mastocytosis, including MCL, exhibit an immature phenotype reflected by the expression of immaturity markers such as CD123, CD34, and HLA-DR and reduced expression of CD117 and FceRI [12]. In our case, although many MCs were morphologically immature (blastoid) looking, they did not express any of the latter immaturity markers (apart from FceRI which is not included in our panel), in addition to a very bright expression of CD117.

Recently, CD30 expression is becoming a widely accepted marker for neoplastic MCs and reported in many cases of MCL (up to 57%). This could have the potential to be a possible therapeutic target for anti-CD30 immunotherapy. Interestingly, CD30 also served as a marker to differentiate neoplastic from benign MCs after allogeneic bone marrow transplantation [13, 14].

### 3.3. CD4 Expression on Neoplastic Mast Cells and Its Suggested Role

CD4 is a marker of T-helper cells. It is also expressed on monocytes, macrophages, and dendritic cells. CD4 expression on normal and neoplastic MCs is not well addressed in the literature, as it had been reported very occasionally on normal MCs mainly in the context of HIV infection [15]. However, several studies have proposed that normal MCs are negative for CD4 [16–18]. Few studies have analyzed CD4 expression in mastocytosis. Although Mirowski et al. [16] reported CD4 expression in frozen sections in cases with cutaneous mastocytosis, Escribano et al. [11] concluded a lack of CD4 expression on MCs. Soilleux and his group had studied CD4 expression on 6 cases of mastocytosis (one SM and 5 cutaneous) after they had encountered a challenging case of CD4-positive cutaneous mastocytosis and found that all the other six cases showed positive mast cell immunostaining for CD4, albeit at slightly lesser staining intensity than small lymphocytes [19].

The actual characterization of CD4 expression on abnormal MCs (particularly in cases of SM) is not well documented and is probably underreported as the previously reported cases rather incidentally detected particularly in cases of cutaneous mastocytosis when CD4 immunostain was performed as part of the panel done to exclude T-cell neoplasms.

It seems reasonable to suggest that a direct cross-talk between T cells and MC populations exists, as recent in vitro studies indicated that MCs may be triggered to degranulate and release cytokines upon heterotypic adhesion to activated T cells [20].

**Figure 5:**
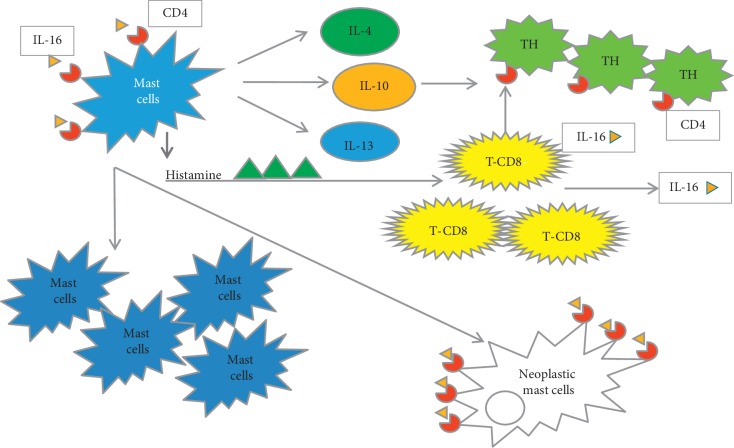
A proposed cartoon showing possible interaction between mast cells and T cells and the role of CD4 molecules. Activated MCs release several cytokines that attract and activate CD4^+^ T cells to become Th2 cells. Histamine may stimulate T-cytotoxic cells to produce interleukin 16 (IL-16), a chemotactic for MCs and CD4-positive T cells. IL-16 acting via CD4 may play a pivotal role in the recruitment, activation, and differentiation of MCs in mastocytosis as well as recruitment of T lymphocytes in mast cell lesions.

Activated MCs release several cytokines that attract and activate CD4+ T cells to differentiate into Th2 cells, inducing humoral immunity [20, 21]. Histamine (produced by MCs) may stimulate T-cytotoxic cells to produce interleukin 16 (IL-16) which is chemotactic for MCs and CD4-positive T cells [22–24]. As CD4 has recently been shown to serve as an IL-16 receptor, thus, it was intriguing to speculate that IL-16 (acting via CD4) plays a pivotal role in the recruitment, activation, and differentiation of MCs in mastocytosis as well as recruitment of T lymphocytes in mast cell lesions [19]. No sufficient data regarding IL-16 in mastocytosis are currently available. A suggested cross-talk between mast cells and T cells is shown in Figure 5.

The role of the CD4 molecule in human monocyte/macrophage function and development is not known. Scott et al. have identified a new function for the CD4 molecule on human peripheral monocytes in triggering the differentiation of these cells into mature functional macrophages, following their interaction with HLA II [25].

Erythrophagocytosis by leukaemic blasts, a poor prognostic feature, is mostly seen in AML with monocytic differentiation particularly those associated with cytogenetic abnormalities involving [8, 16] *t* (p11; p13) and AML with *t* (16; 21) (p11; q22) which is seen in various subtypes of AML. Upon the literature review, we have noticed that CD4 expression on leukaemic blasts had been reported frequently in cases that showed pronounced erythrophagocytosis. Most of these cases are monocytic leukaemias, particularly the rare category of AML with *t* (8; 16). However, it was also reported in T-ALL and in AML with the BCR-ABL1 fusion gene [26–28]. Correlation between CD4 expression (a marker for monocytes/macrophages) on neoplastic MCs and the evident erythrophagocytosis seen in this case needs to be explored further.

### 3.4. Cytogenetic Findings in MCL

No recurrent cytogenetic abnormality was detected in MCL, and a normal cytogenetic profile was found in the majority of cases. In a study performed on 342 consecutive adult patients with SM conducted at the Mayo Clinic between 1976 and 2007 including only 4 patients with MCL showed that the recurrent chromosomal abnormalities in mastocytosis generally included trisomy 8, monosomy 7, del(13q), del(5q), trisomy 10, del(20q), trisomy 6, trisomy 19, and trisomy X, but none of these were detected in MCL [29].

Isolated trisomy 19 detected in our case was previously reported to be strongly associated with myeloid malignancies [30]. A compilation of previously published hematologic neoplasms with trisomy 19 as the sole chromosomal abnormality revealed that the great majority of cases had been myelodysplastic syndromes (MDS) (25 of 31 cases) and fewer cases of myelodysplastic/myeloproliferative neoplasms (MDS/MPN) and AML. Among reported cases of AML with isolated trisomy 19(14 cases), only four cases have had raised on top of MDS, with the trisomy 19 accruing during the time of leukaemic transformation [30]. Up to our best knowledge, trisomy 19 has never been reported in association with MCL. Of note, our case exhibited a background of myelodysplasia; however, mostly, as megaloblastic changes attributed to an associated vitamin B12 deficiency, the possibility of associated MDS cannot be entirely excluded.

## 4. Conclusion

This a report of an extraordinary rare variant of aggressive MCL, with an interesting morphologic, immunophenotypic, and cytogenetic features including hemophagocytosis by neoplastic MCs in addition to the expression of CD4 and CD7 (partially) and trisomy 19. To the best of our knowledge, this is the first case report of CD4-positive MCL. The expression of CD2 and CD25 by abnormal MCs is well documented in the literature; however; expression of other T-cell-associated markers such as CD4 and CD7 was not reported before in ASM or MCL. It is not unexpected given their common lineage that MCs may express some T-cell-associated antigens. Coexpression of CD2, CD4, CD7, and CD30 by the MCs particularly in skin lesions may provoke misinterpretation as a cutaneous T-cell neoplasm.

Currently, insufficient data on CD4 expression in MCL are available in the literature; therefore, the true incidence and significance of its expression remains unknown and requires further investigation. A possible correlation between CD4 expression (a marker for monocytes/macrophages) on neoplastic MCs and the associated erythrophagocytosis seen in this case is probable [31].
